# TransOral Endoscopic Thyroidectomy via Submental and Vestibular Approach: A Preliminary Report

**DOI:** 10.3389/fsurg.2020.591522

**Published:** 2020-11-23

**Authors:** Yilong Fu, Mengwei Wu, Jinbo Fu, Suqiong Lin, Zhengfu Song, Jiyu Chen, Wei Yan, Penghao Kuang, Fusheng Lin, Yezhe Luo, Ende Lin, Xiaoquan Hong, Guoyang Wu

**Affiliations:** ^1^Department of General Surgery, Zhongshan Hospital, Xiamen University, Xiamen, China; ^2^Depatment of General Surgery, Peking Union Medical College Hospital, Chinese Academy of Medical Sciences & Peking Union Medical College, Beijing, China

**Keywords:** thyroid, thyroidectomy, transoral, submental approach, vestibular approach

## Abstract

**Purpose:** Transoral endoscopic thyroidectomy *via* vestibular approach (TOETVA), with its excellent cosmetic effect, has become increasingly popular worldwide. Nonetheless, anatomic obstacles have limited its development to a certain extent. Here, we present our preliminary outcomes of transoral endoscopic thyroidectomy *via* submental and vestibular approach (TOETSMVA), which can overcome those limitations.

**Methods:** From November 2019 to March 2020, we performed TOETSMVA in 21 consecutive patients with thyroid carcinoma at Zhongshan Hospital, Xiamen University. A 1.5-cm lateral incision was made at two fingers below the mandible; two 5-mm incisions were made in the vestibule near the first molars; TOETSMVA was completed through these incisions. The demographic data and surgical outcomes of the patients were retrospectively reviewed.

**Results:** Twenty-one patients with a mean age of 37.5 ± 10.4 years were incorporated into this study. Fourteen patients had papillary thyroid micro-carcinomas, two had papillary thyroid carcinomas, and five had benign nodules. Eight patients had lymph node metastases. All surgeries were performed successfully without conversion to open thyroidectomy. The mean operation time was 138.8 ± 33.2 min; the average hospital stay was 3.3 ± 0.8 days. No patients developed cutaneous paralysis in the midline chin region. Transient recurrent laryngeal nerve paralysis was observed in one patient. There was no evidence of postoperative bleeding, infection, tetany, or other complications.

**Conclusion:** TOETSMVA was shown to be a safe and advisable alternative for selected patients. This approach can overcome the limitations of TOETVA without sacrificing cosmetic results.

## Introduction

Over the past two decades, the global morbidity rate of thyroid carcinoma has sharply increased, and endoscopic thyroid surgery has gained universal favor. The first endoscopic thyroid surgery was performed by Hüscher et al. (1) in 1997. Since then, many studies have evaluated the safety and efficiency of the minimally invasive approach, which moves the anterior cervical incision to other locations [breast (2, 3), axilla (4, 5), or oral cavity (3, 6, 7)]. Obviously, transoral endoscopic thyroidectomy *via* vestibular approach (TOETVA) is the most popular approach, as it provides a short and direct surgical route for thyroid dissection. However, there are some inescapable weaknesses of TOETVA, such as the limitation of large tumor extractions or cutaneous paralysis of the midline chin due to the anatomic obstacles.

Recently, we started to perform transOral endoscopic thyroidectomy *via* submental and vestibular approach (TOETSMVA). The purpose of this article is to determine the feasibility of TOETSMVA, while evaluating whether TOETSMVA can avoid the limitations of TOETVA.

## Materials and Methods

### Patient Selection

We reviewed the demographic data and surgical outcomes of 21 consecutive patients who underwent successful TOETSMVA by the same surgeon (GY Wu) between November 2019 and March 2020. Thyroid nodules, in all patients, were found accidentally by ultrasonography (US) during health examination and were further diagnosed benign nodule, suspicious papillary thyroid cancer (V), or papillary thyroid cancer (VI), by ultrasound-guided fine needle aspiration (FNA). All patients underwent thyroid function, enhanced computed tomography (CT) and electronic laryngoscopy, preoperatively. The eligibility criteria were: (1) a thyroid cancer <2 cm in diameter or a benign nodule <6 cm in diameter; (2) no history of surgical treatment or radiation of the head and neck; (3) no lymph node metastasis or extrathyroidal spread of thyroid cancer according to preoperative examinations. The exclusion criteria were: (1) previous diagnosis of hyperthyroidism or hypothyroidism; (2) infections of the neck or oral cavity; (3) intolerance to general anesthesia, which is inadaptable to surgery.

The patients who intended to seek for the cosmetic effect of surgery were informed of all the advantages and disadvantages of the following techniques: (1) Thyroidectomy *via* Breast Approach; (2) Transoral Endoscopic Thyroidectomy *via* Vestibular Approach, (3) SubMental and Vestibular Approach, and (4) SubLingual and Vestibular Approach. We performed TOETSMVA for 21 qualifying patients who preferred this approach with informed content. The study received approval by the Medical Ethics Committee of Zhongshan Hospital, Xiamen University, Xiamen, Fujian, China.

### Surgical Technique

#### Body Position

When general anesthesia was induced successfully, the patient was placed in the supine position, with both arms adducted and fixed, pillows placed under the shoulders, and the neck slightly extended. The nerve monitoring endotracheal tube was placed *via* the oral route.

#### Prophylactic Antibiotic

A prophylactic antibiotic was administered 30 min before the surgery (8, 9); an additional dose was given if the operation took more than 3 h.

#### Working Space

**Figure 1:**
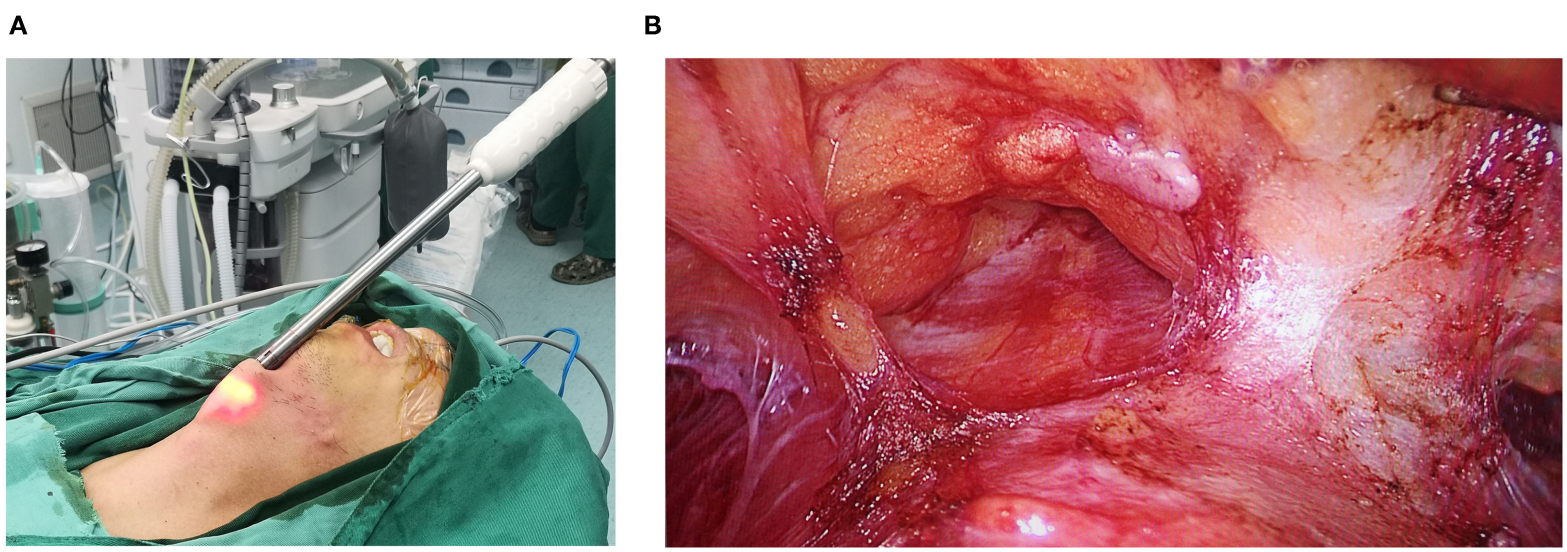
**(A)** The visible subcutaneous stripper; **(B)** The first operative space.

**Figure 2:**
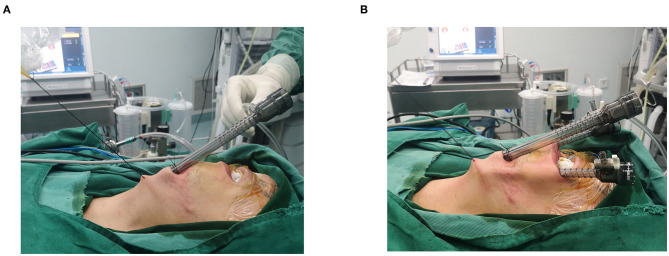
**(A)** CO_2_ insufflation and surgical sutures were used to maintain the operating space; **(B)** Two 5-mm trocars were inserted.

A 1.5-cm lateral incision was made two fingers below the mandible, and the first operative space was established through this incision using the visible subcutaneous stripper (Figure 1). Then, a 10-mm trocar was placed in this incision, CO_2_ insufflation was applied at 4 mmHg with a flow rate of 30 L/min, and surgical sutures were used to further maintain the operating space (Figure 2A). Two 5-mm trocars were then inserted into the oral vestibule, one on each side at the first molar (Figure 2B). For the second operative space, electrocautery and an ultrasound scalpel were used to dissect sharply under the platysma muscle in order to reach the suprasternal fossa on the bottom, and to reach the anterior margin of the sternocleidomastoid on both sides.

#### Thyroid Resection With or Without Central Lymph Node Dissection

**Figure 3:**
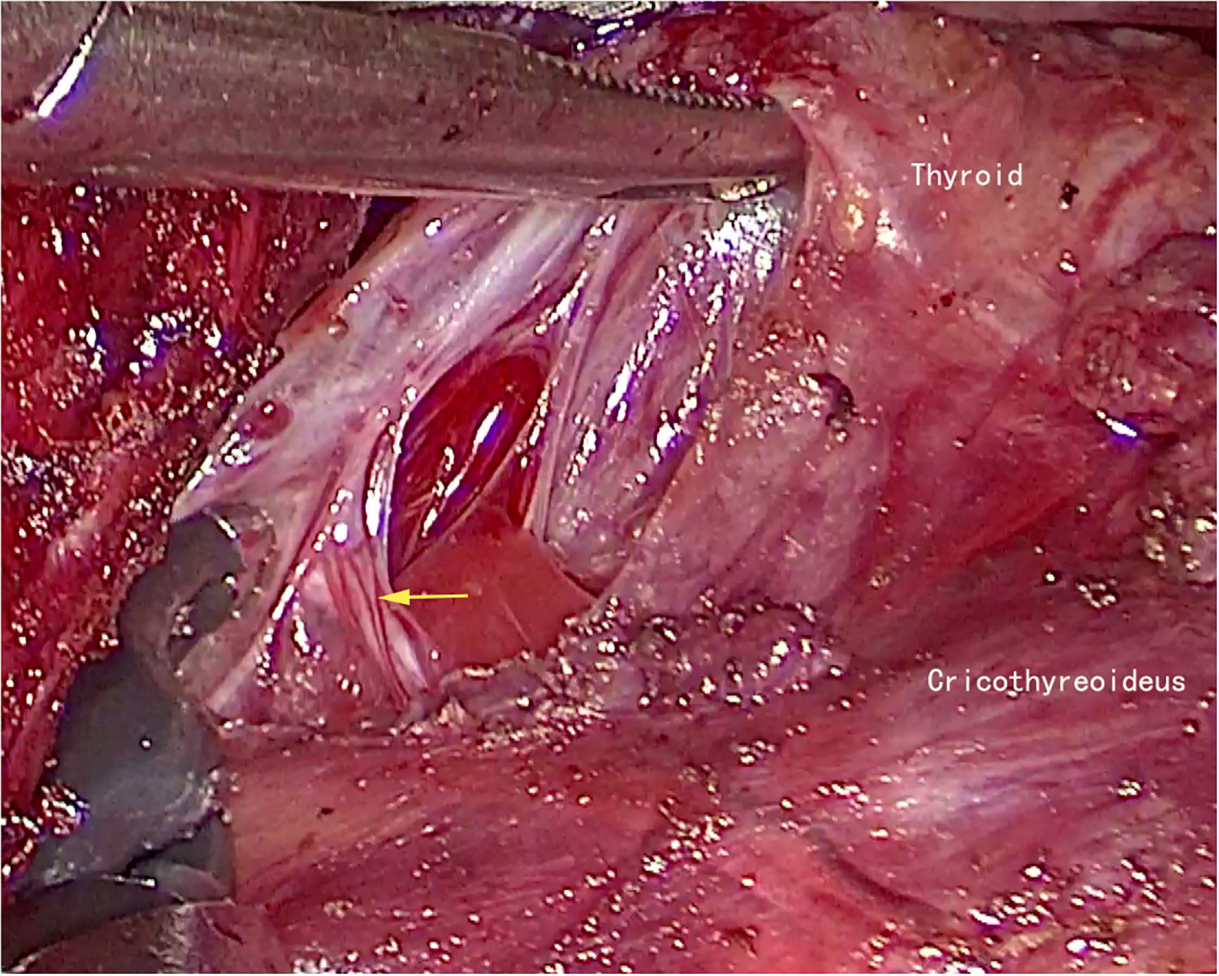
The external branch of superior laryngeal nerve of left side (yellow arrow).

**Figure 4:**
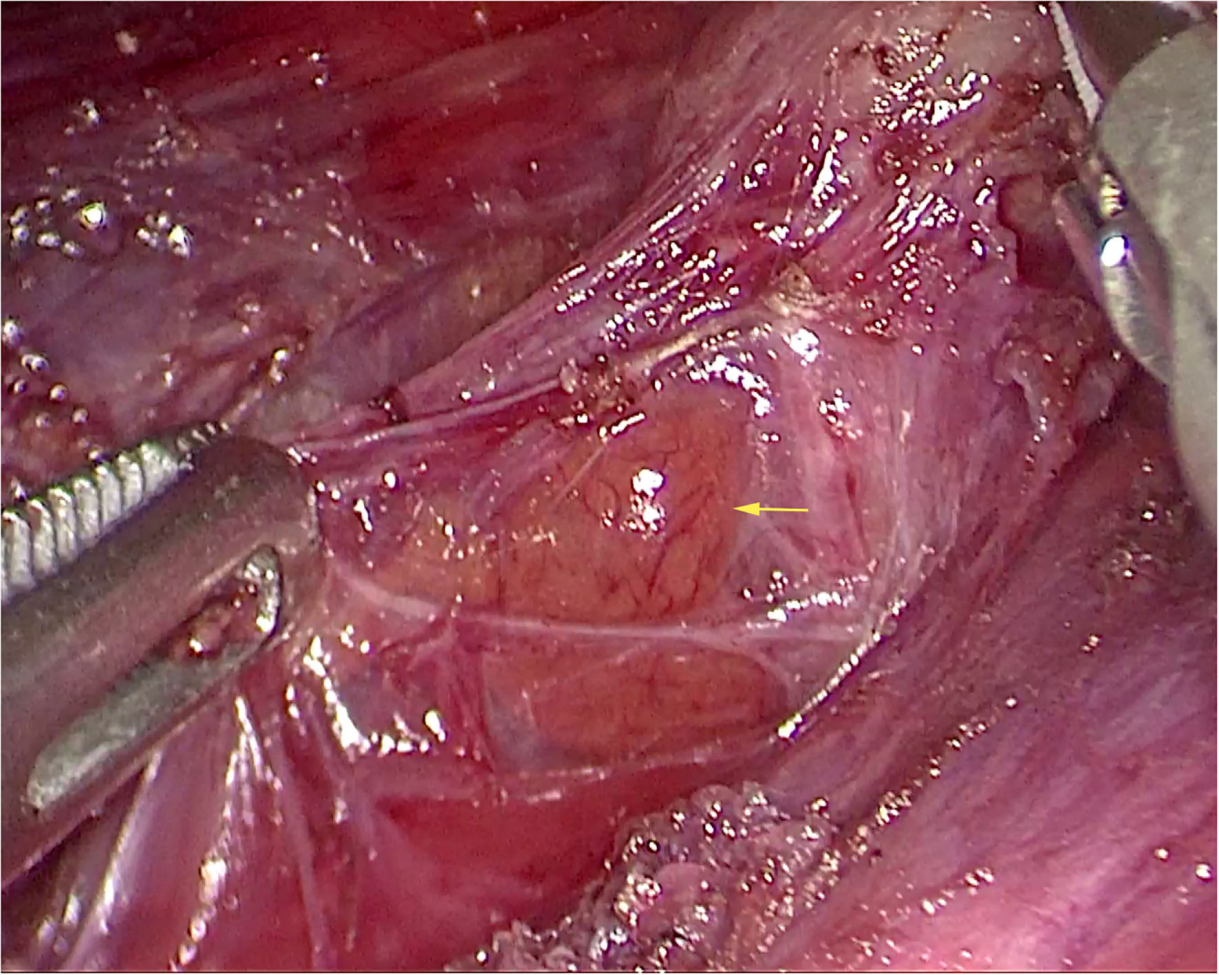
Superior parathyroid gland of left side (yellow arrow).

**Figure 5:**
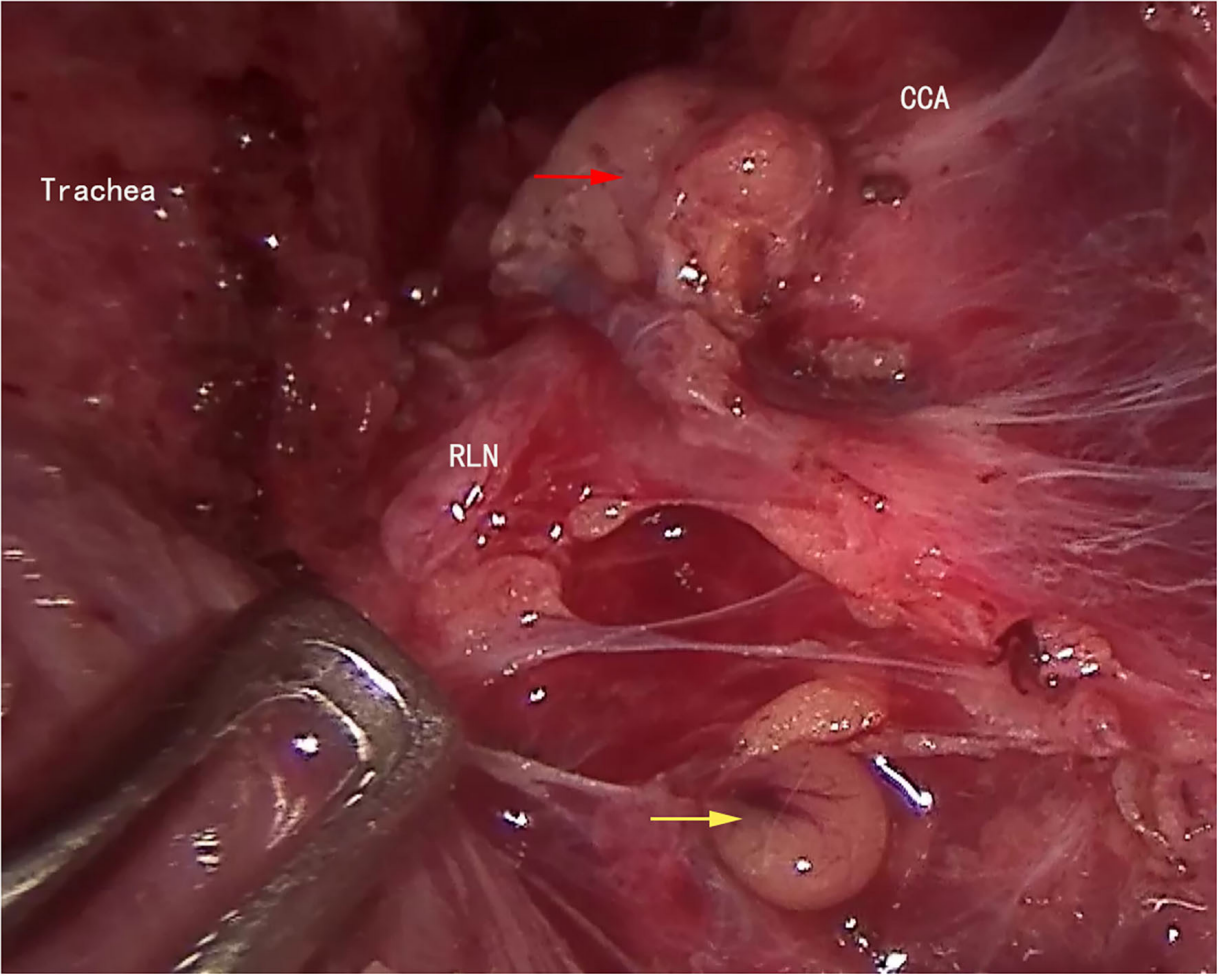
The right side ipsilateral central lymph node dissection was finished. CCA, common carotid artery; RLN, recurrent laryngeal nerve; superior parathyroid gland (yellow arrow); inferior parathyroid gland (red arrow).

All patient underwent the following operations with intraoperative neuromonitoring (IONM). The cervical white line was incised, and the belt-shaped muscle was separated to expose the affected gland. Carbon nanoparticles suspension was then used to dye both the thyroid gland and lymph nodes. To acquire the V1 signal, further separation to reach the carotid sheath was performed with the help of an endoscopic retractor or a surgical suture. Then, using an electrocoagulation hook to remove prelaryngeal lymph nodes, the thyroid isthmus was separated through the anterior tracheal space and was divided using an ultrasound scalpel. The affected thyroid gland was clamped downward to expose the cricothyroid space, the upper thyroid pole was dissected, and the superior thyroid artery was divided by the ultrasound scalpel close to the upper thyroid pole. Carefully identify and protect the external branch of superior laryngeal nerve (EBSLN) (Figure 3). The fine membrane dissection technique was used to protect the superior parathyroid gland (Figure 4) and the recurrent laryngeal nerve (RLN). The R1 signal was acquired by the nerve probe forceps to identify the RLN at its entry point. Dissecting the thyroid gland and Berry's ligament with careful protection of the RLN and the inferior parathyroid gland, the specimens were then removed using endoscopic pouches. Ipsilateral central lymph node dissection was performed prophylactically for patients with papillary thyroid carcinoma (Figure 5). V2 signal and R2 signal were again recoded when thyroid lobectomy and central lymph nodes dissection, of the affected side, were completed.

#### Closing and Postoperative Management

The operative region was washed with 1,000 mL warm saline solution, allowing a check for hemostasis. A surgical drainage tube was placed in the submental incision. The belt-shaped muscle and three incisions were sutured by 3-0 and 4-0 absorbable sutures, respectively. A parathyroid gland that could not be preserved at its native location was transplanted into the left forearm. When the loss of signal (LOS) was diagnosed intraoperatively, the electronic laryngoscopy was performed on day one postoperatively to diagnose vocal cord paralysis, and on every other month after surgery to examine the recovery of the vocal cords.

### Statistical Analysis

SPSS Statistics 22.0 (IBM Inc., Armonk, New York, USA) was used to analyze the medical records of all patients enrolled in this study. Continuous variables were expressed as the mean ± standard deviation, and the groups were compared using the Mann–Whitney *U* test. Categorical variables were compared using the Fisher's exact test. *P-*values <0.05 were considered statistically significant.

## Results

### Demographic Data

Twenty-one patients, 12 female and 9 male, were enrolled in this study. The mean age was 37.5 ± 10.4 years, the mean body mass index was 22.6 ± 2.4 kg/m^2^, the maximum nodule size was 4.9 cm, and the average nodule size was 1.4 ± 1.3 cm. The postoperative pathology results indicated that 14 patients had papillary thyroid micro-carcinomas, 2 had papillary thyroid cancers, and 5 had benign nodules. Lymph node metastases were present in 8 patients (Table 1).

**Table 1:** Demographic data (*n* = 21).

**Variable**	**Value**
**Sex**	
Female	12(57.1%)
Male	9(42.9%)
Age (mean ± SD) (years)	37.5 ± 10.4
BMI (mean ± SD) (kg/m^2^)	22.6 ± 2.4
Nodule size (mean ± SD) (cm)	1.4 ± 1.3
Maximum nodule size (cm)	4.9
**Nodule location**	
Left	11(52.4%)
Right	10(47.6%)
**Pathology**	
Benign nodule	5(23.8%)
PTMC	14(66.7%)
PTC	2(9.5%)
**Lymph node stage in cancer patients**	
N0	8(50%)
N1a	8(50%)

*BMI, body mass index; PTMC, papillary thyroid micro-carcinoma; PTC, papillary thyroid cancer*.

### Surgical Outcomes

**Figure 6:**
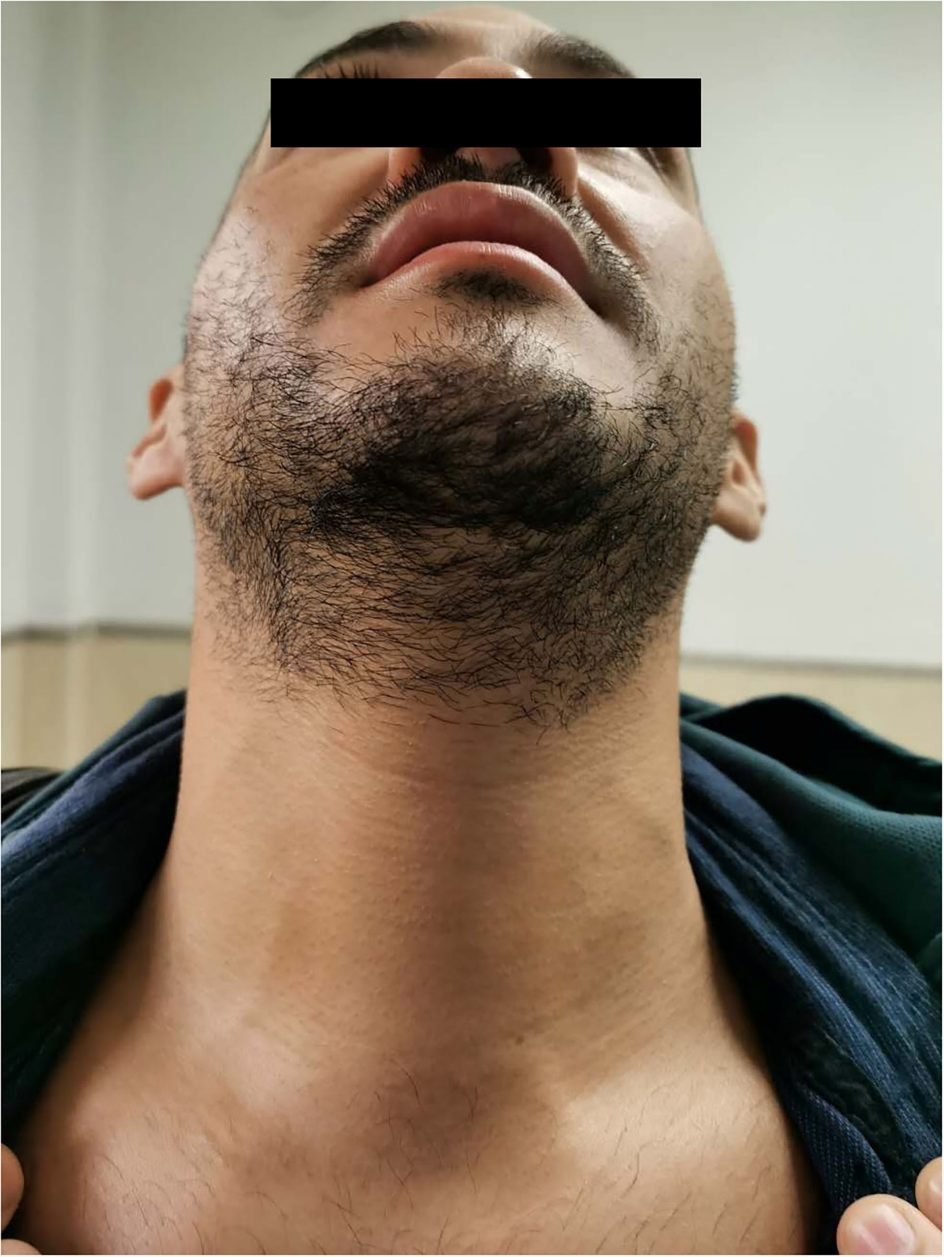
The postoperative picture of 3 months.

All surgeries were performed successfully without conversion to open thyroidectomy. The mean operation time was 138.8 ± 33.2 min and the average hospital stay was 3.3 ± 0.8 days. No patients developed cutaneous paralysis in the midline chin region. Transient RLN paralysis was observed in one patient and it was recovered in two months postoperative (Table 2). There was no evidence of postoperative bleeding, infection, tetany, or other complications. The patients were satisfied with the cosmetic result of this approach (Figure 6).

**Table 2:** Surgical outcomes (*n* = 21).

**Variable**	**Value**
Operation time (min)	138 ± 33.2
Hospital day (days)	3.3 ± 0.8
Intraoperative blood loss (mL)	10.7 ± 6.1
No. of total CLN in cancer patients	5.2 ± 2.7
No. of positive CLN in cancer patients	1.3 ± 2.1
Cutaneous paralysis in the center of chin region	0(0%)
Transient recurrent laryngeal nerve paralysis	1(4.8%)
Permanent recurrent laryngeal nerve paralysis	0(0%)
Postoperative bleeding	0(0%)
Infection	0(0%)
Hypoparathyroidism	0(0%)

*CLN, central lymph node*.

## Discussion

Endoscopic thyroid surgery, which allows for more complex procedures through minimally invasive approaches, has achieved worldwide implementation, particularly in Asia. Among these remote-access surgical options, transoral access was regarded as the most promising approach for thyroid dissection since the first attempt by Witzel et al. (10) in 2008. So far, TOETVA has been the primary option for both surgeons and selected patients. It has inherent advantages, such as scarless surgery with three mucosal incisions, a short direct route for thyroid resection, and an excellent space for central lymph node dissection.

Meanwhile, there are several limitations of TOETVA. First, Zhang et al. (11) reported that there was cutaneous numbness of the lower jaw for 3–12 months; the mental nerves are distributed in the branch-like shape, a 2.0-cm transverse incision in the central oral vestibule is prone to injuring the branch of the mental nerve, resulting in long-term cutaneous paralysis in the midline chin region. However, in TOETSMVA, the observation trocar is moved to the submental region, which reduces the possibility of damage to the mental nerve branch. Second, extrusion of benign thyroid nodules with maximum diameter >4 cm or malignant nodules with maximum diameter >2 cm is limited by the length of the incision and the shape of the mandible; this may require expansion of the central vestibular incision, which increases the probability of mental nerve injury. Third, for patients who underwent previous chin augmentation with implants, the observation trocar and the lever action of the laparoscope can compress or damage the shape and integrity of the chin implants.

In contrast, no patients enrolled in this study developed cutaneous paralysis in the midline chin region; all specimens (maximum diameter of 4.9 cm) were extracted without compromising this function. In TOETVA, the shape of a chin implant was destroyed in one patient at our institution. No patient who underwent TOETSMVA experienced this, but its effectiveness needs to be verified in the future. Even so, TOETSMVA is not applicable for patients who pursue a totally scarless surgical outcome. In fact, the submental area is often used as the surgical incision site for liposuction (12) and platysmaplasty (13) in plastic surgery, since it is hidden from normal view. All 21 patients enrolled in this study were pleased with the cosmetic outcome. Based on our experience, we believe TOETSMVA is more suitable for patients with pointed chin or long mustache.

The learning curve for endoscopic thyroid surgery has been reported by many institutions: 50 cases for the retroauricular approach, 90 cases for transaxillary approach, and 31 cases for the breast approach (14, 15). The learning curve for transoral thyroid surgery was deemed to be shorter than that of other approaches (16). Inabnet et al. (17) claimed that decreasing the amount of dissection needed to create a favorable working space is critical for reducing the operative time of transoral endoscopic thyroidectomy. In our experience, establishing the first operative space is easier in TOETSMVA than in TOETVA, making TOETSMVA more conducive to popularization among abecedarians of transoral thyroid surgery.

In thyroid surgery, transient RLN paralysis occurs in 5–8% of cases, with permanent paralysis in 0.3–3% (18). In one patient of our series, IONM indicated that the signal amplitude decreased by more than 50% during the operation. After investigation, it was diagnosed LOS, which may be type I injury caused by nerve traction. The RLN paralysis was confirmed by electronic laryngoscopy and it was recovered in two months postoperative. We propose the following suggestions, which may contribute to the protection of RLN: (1) use IONM routinely; (2) the functional blade of the ultrasonic knife should back to RLN; (3) the working forceps should be keep away from the RLN more than 3 mm; (4) care should be taken to not overstress RLN, especially when dissecting Berry's ligament.

In conclusion, TOETSMVA was shown to be a safe and advisable alternative for selected patients. This approach can overcome the limitations of TOETVA without sacrificing good cosmetic result. Our study was limited by the short-term follow-up; long-term outcomes and comparison with TOETVA require investigation by further research.

## Data Availability Statement

All datasets generated for this study are included in the article/supplementary material.

## Ethics Statement

The studies involving human participants were reviewed and approved by Zhongshan Hospital, Xiamen University. The patients/participants provided their written informed consent to participate in this study. Written informed consent was obtained from the individual(s) for the publication of any potentially identifiable images or data included in this article.

## Author Contributions

GW: conception and design and study supervision. YF, MW, and JF: development of methodology. SL, ZS, JC, and WY: analysis and interpretation of data. YF, MW, EL, and XH: writing of the manuscript. PK, FL, and YL: review of the manuscript. All authors contributed to the article and approved the submitted version.

## Conflict of Interest

The authors declare that the research was conducted in the absence of any commercial or financial relationships that could be construed as a potential conflict of interest.
